# Population snapshot of Streptococcus pneumoniae causing invasive disease among adults aged ≥18 years in South Africa before and after implementation of pneumococcal conjugate vaccines in 2005–2020

**DOI:** 10.1099/mgen.0.001559

**Published:** 2025-11-19

**Authors:** Kedibone Maria Ndlangisa, Cebile Lekhuleni, Happy Skosana, Linda de Gouveia, Susan Meiring, Sibongile Walaza, Vanessa Quan, Stephen D. Bentley, Stephanie W. Lo, Cheryl Cohen, Anne von Gottberg, Mignon du Plessis

**Affiliations:** 1 National Institute for Communicable Diseases (NICD), a Division of the National Health Laboratory Service, Johannesburg, South Africa; 2 School of Pathology, University of the Witwatersrand, Johannesburg, South Africa; 3 Pathogen and Microbes, Wellcome Trust Sanger Institute, Hinxton, UK; 4 Milner Centre for Evolution, Department of Life Sciences, University of Bath, Bath, UK; 5 Division of Medical Microbiology, Department of Pathology, Faculty of Health Sciences, University of Cape Town, Cape Town, South Africa

**Keywords:** global pneumococcal sequence cluster (GPSC), pneumococcal conjugate vaccine (PCV), pneumococcus

## Abstract

Routine use of pneumococcal conjugate vaccines (PCV) in South Africa caused a decline in vaccine-associated invasive pneumococcal disease (IPD), followed by the emergence of non-PCV serotypes, driven mainly by pre-existing lineages. We determined the molecular epidemiology of isolates causing IPD among adults in South Africa from 2020 to 2025 before and following the implementation of PCV in 2009. We performed whole-genome sequencing on randomly selected isolates causing IPD among adults aged ≥18 years (*N*=1 581) during the four vaccine periods [pre-PCV (2005–2008), PCV7 (2009–2010), early-PCV13 (2011–2014) and late-PCV13 (2015–2020)]. We assigned *in silico* serotype, multi-locus sequence type, clonal complex (CC) and global pneumococcal sequence cluster (GPSC) and determined antimicrobial non-susceptibility profiles *in silico*. Poisson regression was used to calculate incidence rate ratios of imputed individual GPSC lineages using IPD incidence rate estimated per year for the three vaccine periods (PCV7, early-PCV13 and late-PCV13), compared to the pre-PCV period. Overall, our dataset represented 3.7% (*n*=270), 3.4% (*n*=128), 4.9% (*n*=287) and 13.5% (*n*=896) of adult pneumococcal isolates received during the four periods, respectively. We identified 135 GPSCs with the majority of isolates [68.7%(1 086/1 581)] clustering into 1 of 23 dominant GPSCs defined as GPSCs that comprised ≥20 genomes in the dataset. Compared to the pre-PCV7 period, a decrease in incidence of vaccine type lineages normally associated with vaccine serotypes was observed during the late-PCV13 period. GPSC2 (serotype 1) declined from 1.4 to 0.039/100,000 population (*P*<0.001). Some non-PCV lineages increased. GPSC26 (serotype 12F) increased from 0.07 to 0.3 (*P*<0.001). Of the 23 dominant GPSCs, 11 expressed ≥2 serotypes. While the majority of GPSC5/CC172 isolates expressed serotype 23F during the pre-PCV period (61.5%, 7/12), serotype 35B was the most common serotype (57.1%, 12/21) expressed by GPSC5/CC172 isolates during the late-PCV13 period. All GPSC9/CC63 isolates sequenced from the pre-PCV period (*n*=3) expressed serotype 14; however, during the late-PCV13 period, nearly all (88.2%, 15/17) were serotype 15A. The emergence among non-PCV13 serotypes, of lineages usually associated with PCV13 serotypes (such as GPSC5/CC172 and GPSC9/CC63), warrants continued genomic surveillance in South Africa, more so as PCV10 (Pneumosil) replaced PCV13 in South Africa in 2024.

**Impact Statement**

Following the pneumococcal conjugate vaccine (PCV) introduction in South Africa, invasive pneumococcal disease declined significantly among adults predominantly due to decreases in vaccine serotypes. Among isolates from adults, significant increases were noted for some non-vaccine serotypes following PCV introduction. This study compares the population structure of isolates causing invasive pneumococcal disease among adults aged ≥18 years, over a 16-year period spanning years prior to and following the introduction and routine use of PCV7 and PCV13 and before the switch to PCV10 (Pneumosil) in South Africa. We detected emergence among non-PCV13 serotypes, of lineages (such as GPSC5/CC172 and GPSC9/CC63) that are usually associated with PCV13 serotypes. These findings contribute to the general understanding of pneumococcal molecular epidemiology in the PCV era as well as the international effort to characterize replacement serotypes.

## Data Summary

Raw data were deposited in the European Nucleotide Archive (ENA). Individual accessions for the samples are listed in Table S6 (available in the online Supplementary Material).

## Introduction

Pneumococcal conjugate vaccines (PCV) have significantly reduced the incidence of invasive pneumococcal infections due to PCV serotypes among vaccine-eligible children and among unvaccinated children and adults through herd effects [1,2]. Current PCV formulations target specific serotypes, leaving a niche for non-targeted serotypes. Increases in the incidence of invasive pneumococcal disease (IPD) due to non-vaccine serotypes have been observed in some countries following routine use of the PCVs [1,3,6]. These increases have so far been attributed mainly to the expansion of pre-existing non-vaccine serotype clones [7,9].

South Africa introduced PCV7 in 2009 with a three-dose schedule at 6, 14 and 36 weeks of age; this vaccine was replaced (using the same three-dose schedule) with PCV13 in 2011. In 2017, PCV13 (in addition to pneumococcal polysaccharide vaccine) was recommended for use in adults ≥50 years and younger adults ≥18 years with comorbid condition [10]. Among children <2 years of age, IPD incidence due to PCV7 serotypes decreased significantly in 2011 compared to the pre-PCV period [1]. However, a non-statistically significant increase in the incidence of disease caused by non-vaccine serotypes 8 and 15B/C occurred in this age group in 2012 compared to the pre-PCV period [1]. Among adults aged 25–45 years, non-significant increases in non-PCV serotypes 7C, 9N, 10A, 12F, 22F and 34 were observed in 2012 compared to the pre-PCV period [1]. More recent data shows that IPD has declined significantly among adults aged 25–64 years, predominantly due to decreases in vaccine serotypes [11]. Among individuals aged 45–64 years, a significant increase was noted for non-vaccine serotypes in 2019, particularly 8, 12F and 9N [11].

In our previous study, describing the baseline population structure of pneumococcal serotypes prior to routine PCV use in South Africa, predominant lineages, associated with serotypes 3 [sequence type (ST) (ST458)], 4 (ST5410) and 19A (ST2062), differed from those identified globally [12]. Our study on genomic population structure and temporal trends among invasive pneumococci in South African children aged <17 years, prior to and post-PCV implementation, highlighted a decline in lineages predominantly expressing PCV13 serotypes [13]. This current study compares the population structure of isolates causing IPD among adults aged ≥18 years, over a 16-year period spanning years prior to and following the introduction and routine use of PCV7 and PCV13 and before the switch to PCV10 (Pneumosil) in South Africa.

## Methods

### National IPD surveillance (GERMS-SA)

Isolates were collected as part of national, laboratory-based surveillance for IPD in South Africa, initiated in 1999 [14]. In 2003, national surveillance was enhanced to include additional data, such as patient outcome and human immunodeficiency virus serological status from ~30 sentinel sites in all 9 provinces. Isolates and patient data were submitted to the reference laboratory at the National Institute for Communicable Diseases from more than 200 participating private and public laboratories in all 9 provinces. Isolates for this study were from cases reported nationally from 2005 through 2020. A case of IPD was defined as the isolation of *Streptococcus pneumoniae* from a normally sterile-site specimen (e.g. blood, cerebrospinal fluid, pleural fluid and joint fluid). In addition, cases included patients with normally sterile-site specimens which were culture-negative but tested positive by PCR or bacterial latex antigen supported by Gram stain microscopy [15]. The total number of cases included confirmed unreported cases identified through audits of the laboratory information system in participating laboratories. Isolates were received on Dorset egg transport media [Diagnostic Media Products (DMP), Johannesburg, South Africa and Media Mage, Johannesburg, South Africa] and sub-cultured on arrival at the reference laboratory on 5% horse blood agar (DMP) in the presence of an optochin disc (Mast Diagnostics, Virginia, USA) [16]. Pure cultures were stored in 10% skim milk with inositol and glycerol (DMP) at −70 °C.

### Serotyping and antimicrobial susceptibility testing

Serotypes were determined by the Quellung reaction using serotype-specific antisera and pool G antisera for serogroups 29, 34, 35, 42 and 47 (Statens Serum Institute, Copenhagen, Denmark) [17]. Antimicrobial susceptibility testing was performed by agar dilution (for penicillin and ceftriaxone) or Etest (amoxicillin, erythromycin, clindamycin, chloramphenicol, tetracycline, rifampicin, cotrimoxazole, ofloxacin, linezolid and vancomycin) (AB Biodisk, Solna, Sweden) from 2005 through 2008 and by the broth microdilution method using commercially prepared Sensititre-SASP2 panels (Trek Diagnostics Inc., Cleveland, OH) from 2009 onwards. Results were interpreted according to Clinical and Laboratory Standards Institute guidelines and breakpoints [18]. Isolates with minimum inhibitory concentrations ≥0.12 mg l^−1^ were considered non-susceptible to penicillin. For other antimicrobials, isolates were defined as non-susceptible if they were intermediately or fully resistant to the antimicrobial tested. Multidrug non-susceptibility was defined as non-susceptibility to beta-lactams and at least two other classes of antimicrobials.

### Selection of isolates for genetic characterization

Isolates representing all serotypes were randomly selected for sequencing. From 2005 through 2014, selection was stratified to include ~75 isolates from individuals ˃5 years of age for each year. From 2015 through 2020, isolates were stratified to include ~150 isolates from individuals ˃5 years of age for each year. In total, 1,826 isolates from individuals ˃5 years of age were selected for whole-genome sequencing (WGS), of which 1,610 were isolates from adults aged ≥18 years (Table 1). In this study, we analysed 1,581 genomes of isolates from adults; data on isolates from children was previously reported [13].

**Table 1.** IPD cases, viable isolates and isolates with genotyping data, by vaccine period [pre-PCV (2005–2008), PCV7 (2009–2010), early-PCV13 (2011–2014) and late-PCV13 (2015–2020) period], in South Africa

	2005–2008 (*N*, %)	2009–2010 (*N*, %)	2011–2014 (*N*, %)	2015–2020 (*N*, %)	Total (*N*, %)
Number of IPD cases	19,197	8,959	12,625	13,418	54,199
Number of IPD cases with known age	18,328 (95.5)	8,657 (96.6)	11,856 (93.9)	13,076 (97.5)	51,917 (95.8)
Number of IPD cases among adults ≥18 years	10,556 (57.6)	5,522 (63.8)	8,605 (72.6)	10,112 (77.3)	34,795 (67.0)
Viable isolates	7,281 (69.0)	3,768 (68.2)	5,895 (68.5)	6,642 (65.7)	23,586 (67.8)
Isolates with whole-genome sequence results	270 (3.7)	128 (3.4)	287 (4.9)	896 (13.5)	1,581 (6.7)

### Whole-genome sequencing

DNA was extracted using the QIAamp DNA mini kit (Qiagen, Venlo, Netherlands) from bacterial cells pre-lysed as previously described [19]. DNA extracts were quantified using the Qubit instrument and dsDNA BR Assay kit (Life Technologies, Carlsbad, CA, USA). Multiplexed paired-end libraries were prepared using the Nextera XT DNA sample preparation kit (Illumina, San Diego, CA, USA). Genome sequencing was carried out on Illumina HiSeq or NovaSeq platforms at Wellcome Sanger Institute, UK, and on Illumina NextSeq at the NICD Sequencing Core Facility, South Africa.

Genomes were analysed as previously described [20,22]. Briefly, sequencing reads were *de novo* assembled and annotated using an Illumina data pipeline [22]. *In silico* serotypes and multi-locus STs were determined using SeroBA version 1.0.1 and MLSTcheck version 2.1.1706216 [23,24]. Clonal complexes (CCs) were assigned using eBURST and defined as a group of STs with single locus variance within the GPS reference database version 6 (https://www.pneumogen.net/gps/) [20]. Lineages [global pneumococcal sequence cluster (GPSC)] were assigned for each isolate using PopPUNK version 2.4.0 [25]. GPSC5-specific phylogeny was constructed by mapping Illumina reads of GPSC5 isolates against previously prepared lineage-specific references using Burrow-Wheeler Alignment (BWA) version 0.7.17-r1188 [20,26]. Gubbins was used to identify recombination blocks and to create recombination-free phylogenies using the GTR model in RAxML [27,28]. Microreact and Phandango were used for the visualization of the phylogeny and recombination, respectively [28,29]. Penicillin-binding protein (PBP) profiles were assigned based on transpeptidase domain amino acid sequences of 277–359 residues from PBPs 1a, 2b and 2x with three-number combination PBP genes referred to with standard nomenclature (*pbp*1a-*pbp*2b-*pbp*2x) [21].

A vaccine-type (VT) lineage was described as any GPSC where at least 50% of isolates from the pre-PCV period expressed PCV13 serotypes (1, 3, 4, 5, 6A, 6B, 7F, 9V, 14, 18C, 19A, 19F and 23F) and non-vaccine-type (NVT) lineage as any GPSC where at least 50% of isolates pre-PCV expressed non-PCV13 serotypes.

### Statistical analysis

The study population was corrected for sampling framework by year and by age group using selected sample proportions against the GERMS-SA database. The overall incidence of lineages was calculated using imputed proportions as previously described [13] by assuming that the proportion of lineages by year and age group as determined through WGS was similar to that of the total number of IPD cases identified through surveillance for each year and age group. Poisson regression was used to calculate incidence rate ratios (IRRs) at 95% confidence interval (CI) of imputed individual GPSC lineages using IPD incidence rate estimated per year for the three vaccine periods (PCV7, early-PCV13 and late-PCV13), compared to the pre-PCV period. Mid-year population statistics for South Africa for the three vaccine periods were used as denominators to calculate IPD incidence rate estimates [30]. To correct for over-dispersion, if observed, the robust standard error for minor violation of the Poisson regression assumption measured by goodness-of-fit at *P* value ranging between 0.01 and 0.05 was reported, or the negative binomial regression measured at Poisson goodness-of-fit *P* value <0.01 was used. If none of the models fit, IRR was calculated using the average annual incidence for the three vaccine periods. To avoid IRR equating to zero or infinity, a constant number of one was added to all estimated IPD cases for when a GPSC was not observed in any of the three vaccine periods. Statistical significance was at <0.05 significance level using a two-sided *P* value. Benjamini–Hochberg false discovery rate of 5% was used to correct for multiple testing when the number of tests exceeded ten. A generalized linear binomial regression was used to detect changes in antimicrobial non-susceptibility to penicillin, erythromycin, clindamycin, chloramphenicol, tetracycline, co-trimoxazole and multidrug resistance (MDR) between the pre-PCV and late-PCV13 period. We define MDR as non-susceptible to three or more classes of antibiotic. All statistical analyses were performed in R version 4.2.1. R scripts were adapted from a public repository (https://github.com/rgladstone/GPSCs/tree/master/lo_et_al).

## Results

### National IPD surveillance and serotype distribution

**Fig. 1.**
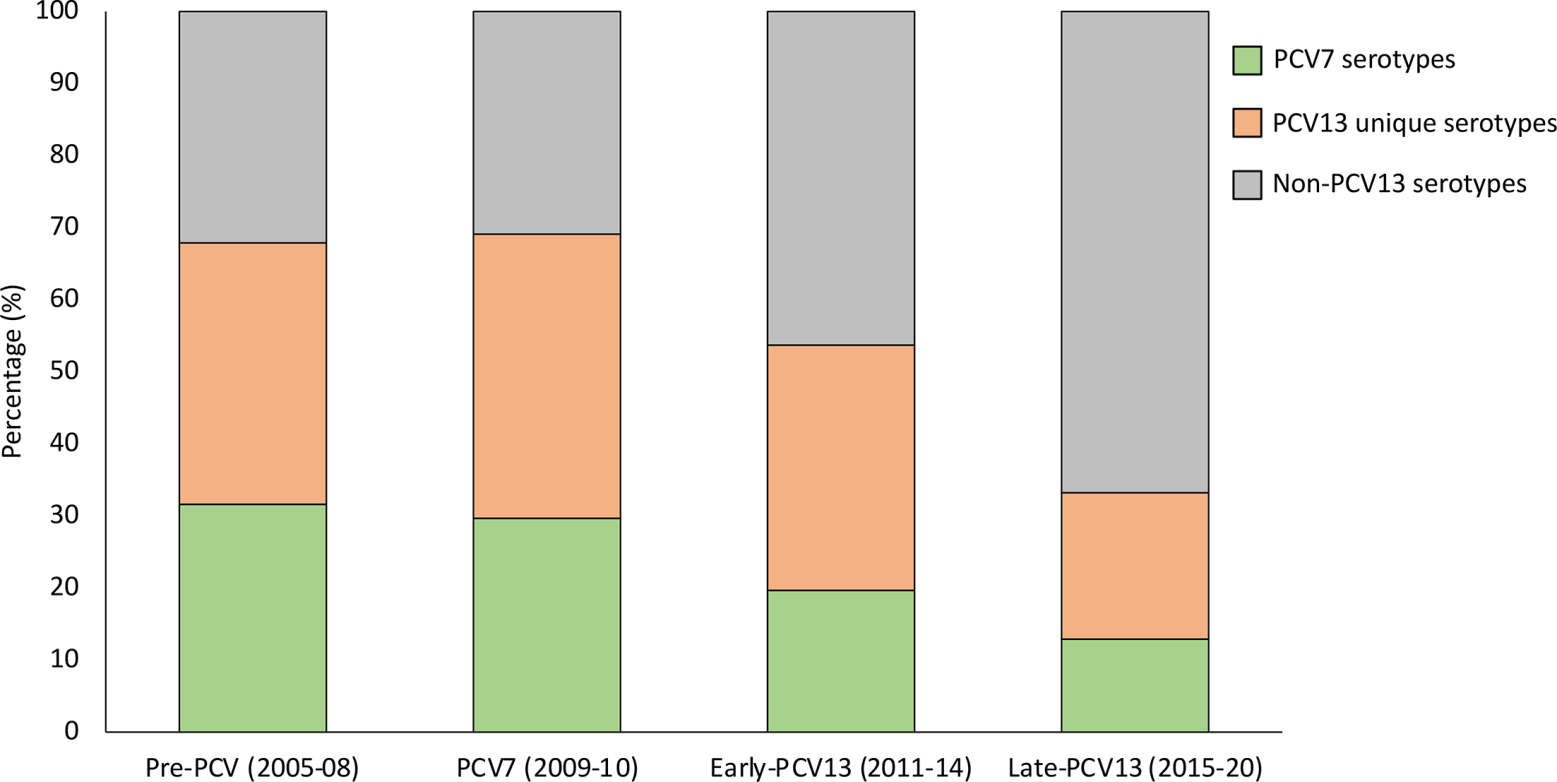
Percentages of invasive pneumococcal diease cases among adults ≥18 years old in South Africa by vaccine period and serotypes. The following numbers were used as denominators for percentage calculations: 7,281 (pre-PCV), 3,768 (PCV7), 5,895 (early-PCV13) and 6,642 (late-PCV13). Serotypes were grouped in PCV7 serotypes (4, 6B, 9V, 14, 18C, 19F and 23F), additional PCV13 serotypes (1, 3, 5, 6A, 7F and 19A) and non-PCV13 serotypes.

**Fig. 2.**
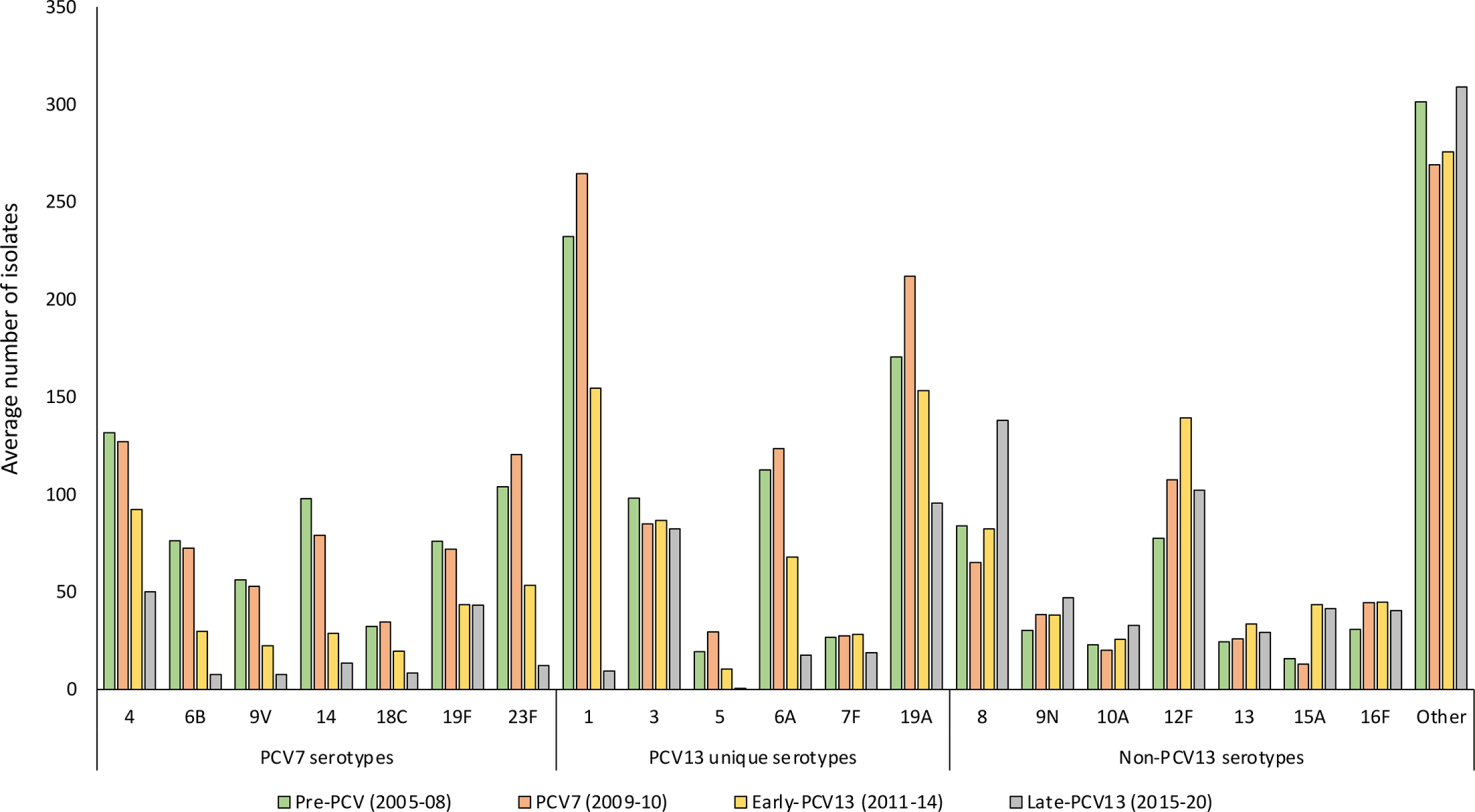
Average number of isolates from invasive pneumococcal specimens of adults ≥18 years old in South Africa by vaccine period and serotype.

From 2005 through 2020, 54,199 IPD cases were reported through GERMS-SA in South Africa (Table 1). Individuals aged ≥18 years represented 67.0% (34,795/51,917) of IPD cases with recorded age. The majority of the cases were reported from four of the nine provinces in South Africa [Gauteng (14,661/34,795, 42.1%), Western Cape (6,671/34,795, 19.2%), KwaZulu-Natal (3,558/34,795, 10.2%) and Eastern Cape (3,105/34,795, 8.9%)]. The percentage of reported cases from the remaining five provinces ranged from 1.8% to 6.2%. Of the 34,795 adult IPD cases, a total of 10,556, 5,522, 8,605 and 10,112 IPD cases were reported during pre-PCV (2005–2008), PCV7 (2009–2010), early-PCV13 (2011–2014) and late-PCV13 (2015–2020) periods, respectively. Viable isolates were available for 7,281 (69.0%), 3,768 (68.2%), 5,895 (68.5%) and 6,642 (65.7%) of cases from the four periods, respectively. The majority of available isolates were from Gauteng (9,762/23,586, 41.4%), Western Cape (5,560/23,586, 23.6%), KwaZulu-Natal (2,361/23,586, 10.0%) and Eastern Cape (1,759/23,586, 7.5%). The percentage of isolates from the remaining provinces ranged from 1.9% to 6.1%. The percentage of PCV13 serotype disease declined from 67.8% (4,933/7,281) during the pre-PCV period to 33.2% (2,202/6,642) during the late-PCV13 period (Fig. 1). Serotypes 8, 12F 16F and 9N were the most common non-PCV13 serotypes overall across all periods and had higher proportions during the late-PCV13 period compared to the pre-PCV period (Fig. 2). Serotypes 19A and 3 were the most common PCV13 serotypes during the late-PCV13 period and ranked third and fourth, respectively (after serotypes 8 and 12F) among serotypes causing IPD during this period.

### Genomic population structure

Of the 1,610 isolates that were sequenced, we identified 39 isolates (2%) with discordant serotype results. Among the 39 isolates, 10 were still viable and both Quellung and WGS results were concordant after repeat testing. The remaining 29 could not be repeated due to loss of viability and were therefore excluded from this analysis. In total, 1,581 isolates with concordant serotype results were included in the study. WGS resolved serotypes for 27 isolates that initially reacted with pool G serogroup antisera and could not be differentiated to serotype level by Quellung [29 (*n*=1), 34 (*n*=9), 35A (*n*=2), 35B (*n*=12) and 35F (*n*=3)]. The 1,581 isolates with WGS data included mainly isolates from Gauteng (542/1,581, 3.4%), Western Cape (499/1,581, 3.2%), KwaZulu-Natal (8.2%) and Eastern Cape (8.7%). Overall, 1,581 genomes represented 3.7% (*n*=270), 3.4% (*n*=128), 4.9% (*n*=287) and 13.5 % (*n*=896) of all pneumococcal isolates collected from adults during the pre-PCV, PCV7, early- and late-PCV13 periods, respectively (Table 1).

**Fig. 3.**
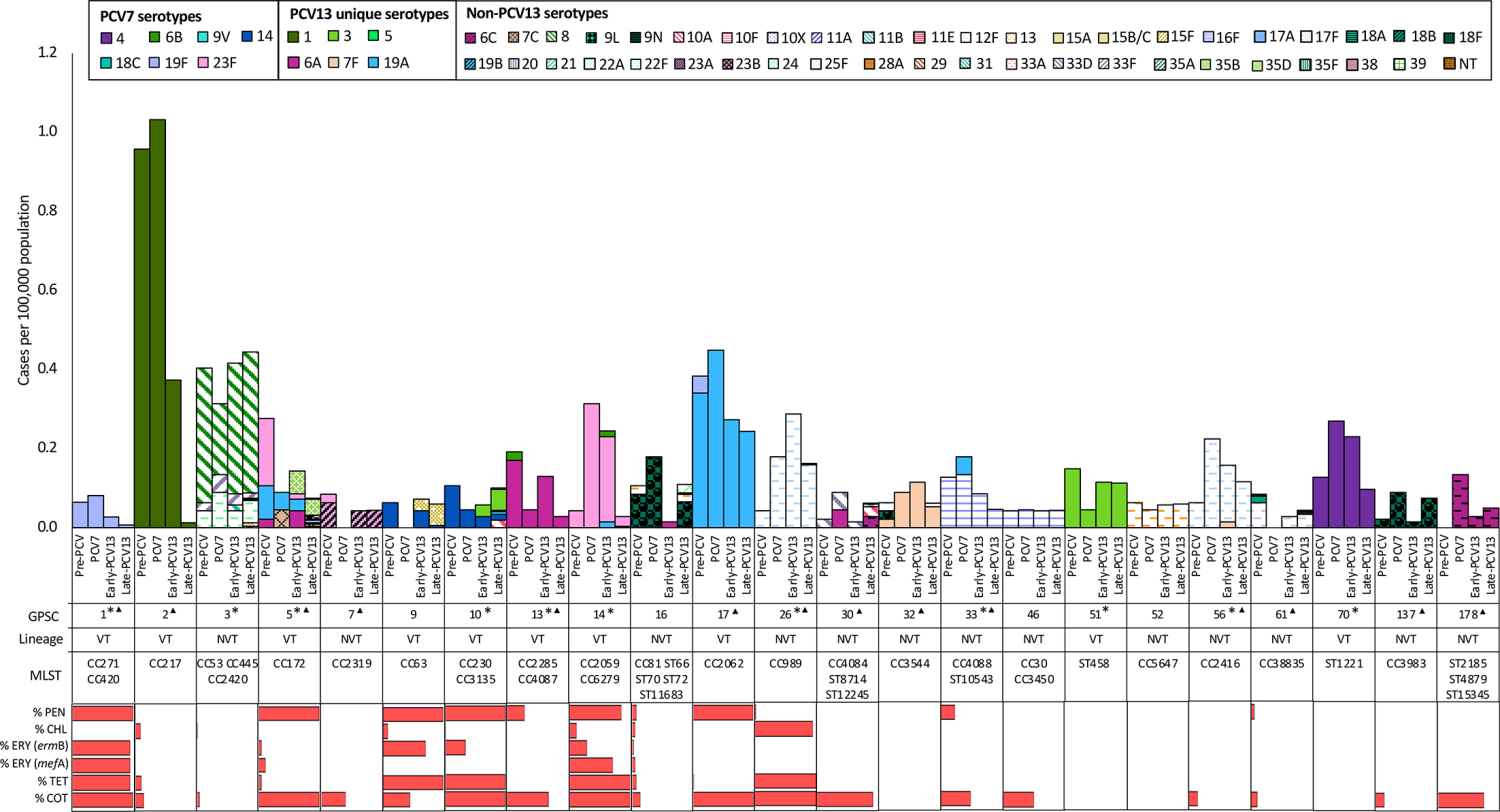
Dynamics of GPSCs among isolates causing IPD among adults ≥18 years old in South Africa during the pre-PCV (2005–2008), PCV7 (2009–2010), early-PCV13 (2011–2014) and late-PCV13 (2015–2020) periods. Only common GPSCs that had at least 20 isolates detected for the entire study period (2005–2020) are shown on the graph. Cases per 100,000 population are plotted by GPSC with stratification into vaccine periods and multilocus sequence typing CC/ST. Bars are coloured by serotypes and solid fill represents PCV13 serotypes, while patterns represent non-PCV13 serotypes. Significant changes in the average incidence between the pre-PCV and PCV7 or late-PCV13 periods are represented by an asterisk or triangle, respectively. Significance was determined at <0.05 using a two-sided *P* value where applicable. The antibiotic resistance pattern to penicillin, chloramphenicol, erythromycin, co-trimoxazole, tetracycline and MDR is presented for each GPSC and in the entire study period.

**Fig. 4.**
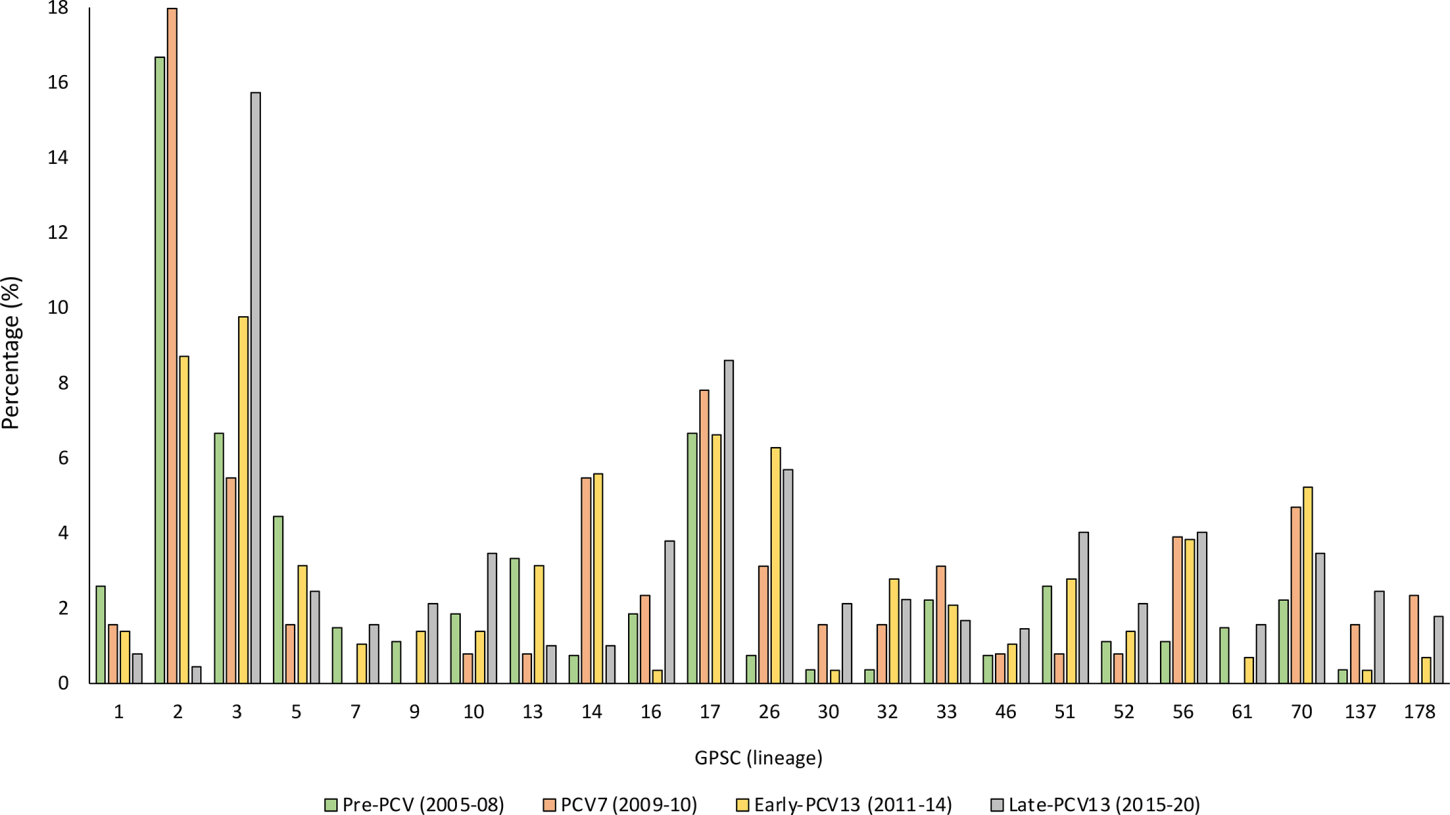
Percentages of common GPSCs among isolates causing IPD among adults ≥18 years old in South Africa during the pre-PCV (2005–2008), PCV7 (2009–2010), early-PCV13 (2011–2014) and late-PCV13 (2015–2020) periods. Common GPSCs (*n*=23) that had at least 20 isolates detected for the entire study period (2005–2020) are shown on the graph. The following numbers were used as denominators for percentage calculations: 270 (pre-PCV), 128 (PCV7), 287 (early-PCV13) and 896 (late-PCV13).

A total of 135 GPSCs were identified and the majority of isolates [68.7% (1,086/1,581)] were distributed among 23 GPSCs. Each of these 23 dominant GPSCs comprised ≥20 genomes in the dataset (Fig. 3). The five most prevalent lineages were GPSC2, 3, 17, 26 and 70 (Fig. 4). GPSC2 (CC217, serotype 1) was the most common lineage during the pre-PCV and PCV7 periods, accounting for 16.7% (45/270) and 18.0% (23/128) of sequenced isolates for these two vaccine periods, respectively. This lineage ranked second during the early-PCV13 period (25/287, 8.7%) and was represented by 0.4% (4/896) of the genomes during the late-PCV13 period. During the early and late-PCV13 periods, GPSC3 (CC53, serotypes 8 and CC445, serotype 22F) was the most common lineage and accounted for 9.8% (28/287) and 15.7% (141/896) of sequenced isolates for the two periods, respectively. GPSC3 ranked second during the pre-PCV period (18/270, 6.7%). During both the PCV7 and late-PCV13 periods, GPSC17 (CC2062, serotype 19A) was the second most common and accounted for 7.8% (10/128) and 8.6% (77/896) of sequenced isolates from the two periods, respectively. While GPSC26 (CC989, serotype 12F) was the third most common lineage during the late-PCV13 period (51/896, 5.7%), during the pre-PCV and PCV7 period, GPSC17 and GPSC14 (CC2259 and CC6279, serotype 23F) were the third most common lineages (18/270, 6.7% and 7/128, 5.5%, respectively).

We assessed changes in incidences of individual lineages between the pre-vaccine period and each of the three vaccine periods (PCV7, early-PCV13 and late-PCV13). Significant declines in average incidences were observed across all three vaccine periods for VT lineages (Fig. 3, Tables S1–S3). For example, for GPSC2 (serotype 1), average incidence/100,000 population declined from 1.4 pre-PCV to 0.039 during the late-PCV13 period GPSC2 (IRR 0.02, 95% CI 0.37–0.011, *P*<0.001). Increases were observed for some NVT lineages. GPSC26 (serotype 12F) increased from 0.067 pre-PCV to 0.25 (IRR 3.67, 95% CI 2.29–4.89, *P*<0.001).

### GPSCs expressing ≥2 serotypes

**Fig. 5.**
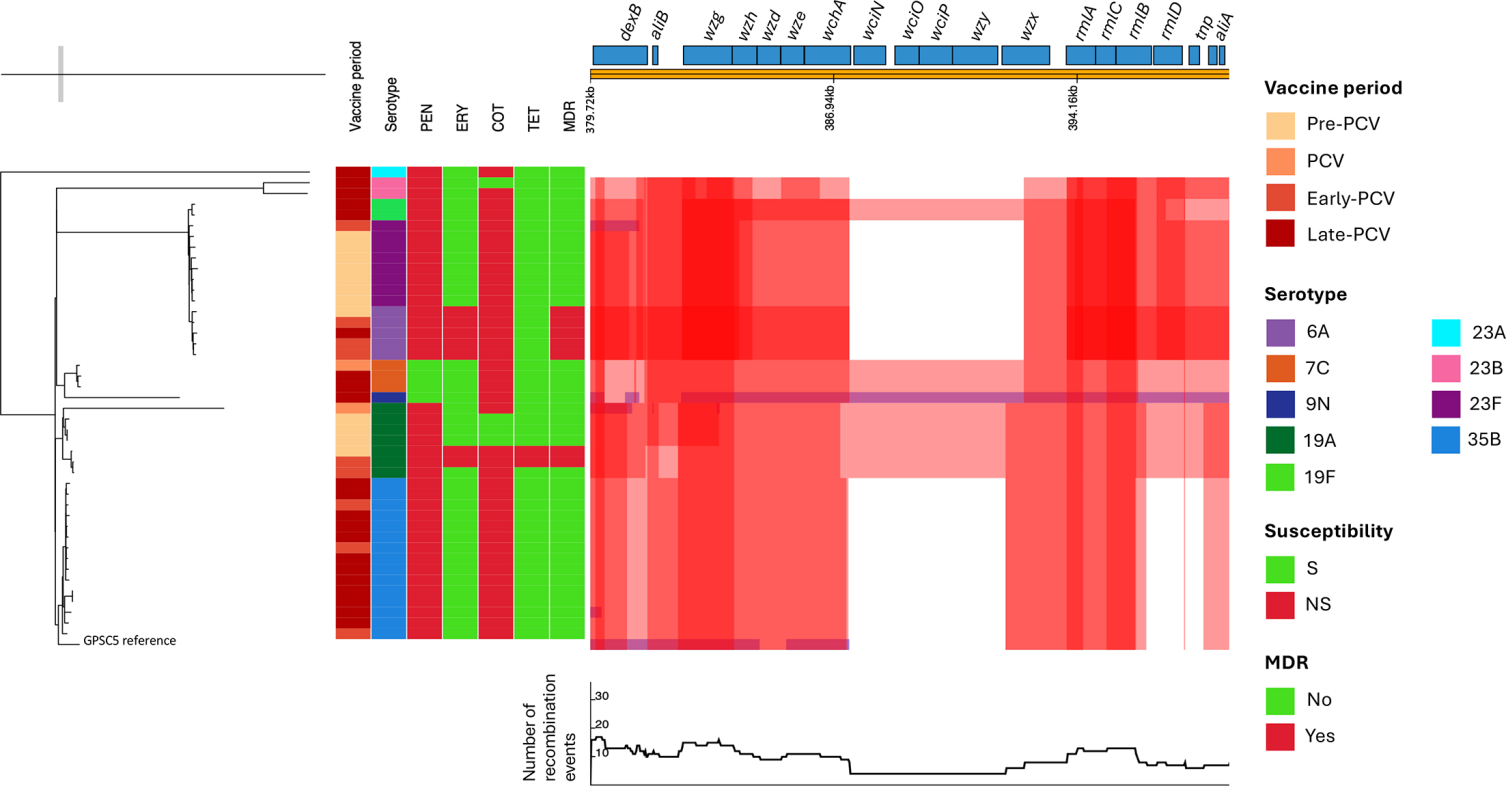
Phylogeny of GPSC5 pneumococcal isolates from South African adults aged ≥18 years. This phandango plot of recombination detected with Gubbins focuses on the cps locus. The top panel indicates positions and annotations of genes and the middle panel shows recombination blocks where red blocks indicate the recombination shared by the corresponding clade. The number of recombination events is shown in the lower panel.

Among the 23 common GPSCs, 11 expressed 2 or more serotypes (Table S4). Of the 11 GPSCs, 6 (GPSC5, GPSC9, GPSC10, GPSC13, GPSC14 and GPSC17) were classified as VT lineages (i.e. at least 50% of the isolates that were isolated during the pre-PCV period expressed PCV13 serotypes). Among GPSC5 and GPSC9, during the pre-PCV period, we did not identify any isolates expressing non-PCV13 serotypes; however, during the late-PCV13 period, the majority (19/22, 86.4% and 15/17, 88.2%, respectively) expressed non-PCV13 serotypes (Table S4). While serotype 23F was the most common serotype within GPSC5 during the pre-PCV period (7/12, 58.3%), during the late-PCV13 period, serotype 35B was the most common serotype (54.5%, 12/22). During the pre-PCV period, none of the GPSC5 isolates expressed serotype 35B. Similarly, none expressed 23F during the late-PCV13 period. To investigate the relationship between isolates belonging to the different serotypes within GPSC5, we constructed a GPSC5-specific phylogeny. Even though GPSC5 isolates clustered according to serotype, except for one isolate belonging to serotype 23A, there was evidence of recombination for most of the capsular locus genes (Fig. 5). All GPSC9 isolates from the pre-PCV period (*n*=3) expressed serotype 14. On the contrary, a smaller proportion of GPSC9 isolates expressed serotype 14 during the late-PCV13 period (2/17, 11.8%). During the late PCV13 period, the majority of GPSC9 isolates expressed serotype 15A (15/17, 88.2%).

### Antimicrobial susceptibility

For the 1,581 isolates in our dataset, overall, there were no significant differences in the prevalence of isolates with antibiotic non-susceptibility when comparing the pre-PCV and late-PCV13 periods (Table 2). Among non-PCV13 serotypes, significant increases were observed for penicillin (3.3%–12.4%, *P*=0.02), chloramphenicol (2.2%–8.3%, *P*=0.05), tetracycline (5.5%–15.5%, *P*=0.01) and co-trimoxazole (27.5%–39.1%, *P*=0.04). In addition, there were more MDR non-PCV13 isolates during the late-PCV13 period compared to the pre-PCV period (13.7% vs. 2.2%, *P*=0.006). Antimicrobial susceptibility profiles of common lineages are shown in Fig. 3 and Table S5. GPSC1 (CC271/CC420, serotype 19F) had the highest percentage of isolates with antibiotic non-susceptibility. All (*n*=20) were resistant to penicillin and co-trimoxazole, with 95% resistant to tetracycline and erythromycin. All isolates belonging to GPSC9 [CC63 (serotype 14 and 15A)] and GPSC10 [CC230 (serotype 14), CC3135 (serotype 10A) and ST700 (serotype 3)] (*n*=26 and 42, respectively) were resistant to penicillin and tetracycline. In addition, all GPSC10 isolates were resistant to co-trimoxazole. The majority (70.7%, 18/26) of GPSC9 isolates were also resistant to erythromycin, and over 40% (12/26) were resistant to co-trimoxazole. Most of the GPSC14 isolates [CC2059 (serotype 23F) and CC6279 (serotype 23F)] were resistant to penicillin (85.3%, 29/34), erythromycin (100%), tetracycline (100%) and co-trimoxazole (100%). The majority of GPSC17 (serotype 19A) isolates were resistant to penicillin (97.6%, 121/124) and co-trimoxazole (98.4%, 122/124). Among NVT lineages, GPSC26 had the highest percentage of antimicrobial non-susceptibility (96% for chloramphenicol, 100% for tetracycline and co-trimoxazole). Penicillin non-susceptibility was low among GPSC26 isolates (1%, 1/77).

**Table 2.** Changes in the prevalence of antibiotic non-susceptible *S. pneumoniae* isolates among adults ≥18 years old in South Africa, comparing the pre-PCV (2005–2008) and the late-PCV13 (2015–2020) periods

	All serotypes			Non-PCV13 serotypes only		
Antibiotic	Pre-PCV (*N*=270)	Late PCV13 (*N*=896)	*P* value using linear regression	Pre-PCV (*N*=91)	Late PCV13 (*N*=611)	*P* value using linear regression
Penicillin, *n* (%)	61 (22.6)	245 (27.3)	0.4	3 (3.3)	76 (12.4)	0.02
Erythromycin, *n* (%)	18 (6.7)	69 (7.7)	0.9	0 (0)	31 (5.1)	1.0
Clindamycin, *n* (%)	16 (5.9)	43 (4.8)	0.5	0 (0)	21 (3.4)	1.0
Chloramphenicol, *n* (%)	7 (2.6)	54 (6.0)	0.05	2 (2.2)	51 (8.3)	0.05
Tetracycline, *n* (%)	34 (12.6	152 (17.0)	0.1	5 (5.5)	95 (15.5)	0.01
Co-trimoxazole, *n* (%)	111 (41.1)	380 (42.4)	0.7	25 (27.5)	239 (39.1)	0.04
MDR*, *n* (%)	32 (11.9)	137 (15.9)	0.1	2 (2.2)	84 (13.7)	0.006

Only isolates with genotypic data were used for calculations..

*MDR was defined as non-susceptible to three or more classes of antibiotics.

## Discussion

We describe the population structure of isolates causing IPD over a 16-year period, from 2005 to 2020, in South Africa, among adults aged ≥18 years. During this period, PCV7 and PCV13 were introduced in the routine infant immunisation schedule in 2009 and 2011, respectively. Among this adult population, percentages of PCV13 serotypes declined during the PCV13 period compared to the pre-PCV period. Percentages of non-PCV13 serotypes 8, 12F and 15A increased. Over 130 GPSCs were identified among sequenced isolates, with the majority of IPD caused by 22 lineages. VT lineages (e.g. GPSC2, serotype 1) declined and some NVT lineages increased (e.g. GPSC26, serotype 12F). VT lineages GPSC1, 9, 10, 14 and 17 had the highest percentage of isolates with antimicrobial non-susceptibility. Among NVT lineages, GPSC26 had the highest percentage of antimicrobial non-susceptibility.

While PCV13 serotypes caused nearly 70% of IPD cases during both the pre-PCV and PCV7 periods, they were responsible for a lower proportion of IPD cases during the early and late PCV13 periods (54% and 33%, respectively). This was reflected in the genotypic data in that, during the pre-PCV and PCV7 periods, GPSC2, a lineage associated with PCV13 serotype 1 predominated and then declined following PCV13 implementation, while during the early-PCV13 and late-PCV13 periods, GPSC3 predominated. GPSC3 is associated with serotype 8, an important non-PCV13 serotype that significantly increased among adult IPD cases during the PCV13 era in South Africa [11]. This lineage has previously been reported as the most common lineage among NVT serotypes, causing IPD in children during the PCV13 period in Europe, the USA and Japan [31]. GPSC26, another lineage associated with non-PCV13 serotype 12F that increased following PCV introduction among South African adults [11], was also common during the early and late-PCV13 periods. GPSC26 is a major lineage among 12F isolates globally [31].

Compared to the pre-PCV period, we did not observe much variation in the incidence of lineages for the PCV7 and early-PCV13 periods, presumably because it was too soon after vaccine implementations. For lineages where significant decreases in incidence were observed during one or both of these vaccine periods, changes could be explained by their association with vaccine serotypes, namely, serotypes 1 (GPSC2), 6A (GPSC5 and 13), 9V (GPSC54) and 23F (GPSC5).

Compared to the pre-PCV period, during the late-PCV13 period, we detected significant variations in incidences of 11 lineages, including significant increases in four lineages. These increases are not unexpected since all four lineages are associated with non-PCV13 serotypes 12F (GPSC26 and 56), 7F (GPSC92) and 9N (GPSC137) that have increased in incidence among adult IPD cases in South Africa [1,11].

In our previous study analysing the GPSC5 lineage among children and adult IPD cases in South Africa from 2005 to 2014, we demonstrated the switch in serotype predominance in this lineage from PCV serotype 23F pre-PCV to 35B during the PCV13 era [32]. This trend is sustained as our current dataset includes genome data from more recent years. This is also similar to our recent data for children in South Africa [13]. We also saw a change in serotype prevalence for GPSC9 from VT 14 to NVT 15A between pre-PCV and late-PCV13 periods. The detection of GPSC9 among both serotype 14 and 15A isolates is not unexpected. GPSC9 is a globally disseminated multidrug-resistant lineage (also known as Sweden^15A^-25) commonly associated with serotype 15A but also detected among serotype 14 isolates [33,35]. Similar to our study, the increase in prevalence of GPSC9 isolates expressing serotype 15A following vaccine intervention was reported in pneumococci isolated from Nepalese children [33]. This lineage is also predominantly expressed by 15A isolates in the USA, Israel and China, post-PCV [36].

Serotypes 3 and 19A are the most common PCV13 serotypes circulating during the late-PCV13 period in South Africa. As previously demonstrated by our earlier study on population structure of pneumococcal serotypes prior to routine PCV, predominant lineages for both of these serotypes are not as common in other parts of the world. While GPSC12 (CC180) is the main serotype 3 lineage globally, in South Africa, GPSC51 (CC458) is the main lineage before and after PCV implementation. Unlike in other countries where increases in 19A are commonly mediated by GPSC1 (CC320) (which was not detected among our 19A isolates), GPSC17 (CC2062) is responsible for the persistence of 19A among adults and children in South Africa [13].

We identified five lineages [GPSC1 (19F), GPSC5 (mainly 35B, 23F, 19A and 6A), GPSC9 (14 and 15A), GPSC10 (mainly 14, 3 and 10A), GPSC14 (mainly 23F) and GPSC17 (19A)] that had high proportions of isolates resistant to penicillin. All six are globally disseminated antibiotic-resistant lineages that are mainly associated with PCV13 serotypes and include GPSC5 and GPSC9 which increased in South Africa among non-PCV13 serotypes following PCV implementation [20,31, 34, 35].

We recently published data on IPD isolates collected from children in South Africa during the same period as this current dataset [13]. Key findings were similar for data from children and adults. Overall, dominating lineages were the same (GPSC2, 3 and 17) and in both datasets, some VT lineages (GPSC2, serotype 1) declined during the late PCV13 compared to the pre-PCV period, with expansion of certain NVT (e.g. GPSC3, serotype 8). In both datasets, the predominating serotype for GPSC5 shifted from 23F to 35B and within GPSC9 from serotype 14 to 15A, during the pre-PCV compared to the PCV13 period.

A limitation of our surveillance system is that because it is laboratory-based, variations in specimen-taking practices and submission of isolates for serotyping might lead to missing data. Another limitation is the small sample size of sequenced isolates (7% of isolates submitted for IPD surveillance in adults in South Africa during this study period). We addressed this through imputation where isolates were unavailable; however, this could have potentially decreased our power to detect uncommon lineages. The pneumococcal population structure presented here may therefore not be an accurate reflection of all lineages circulating in this population. Nevertheless, predominating lineages before and after PCV13 implementation correlate with data on serotypes circulating in South Africa. For example, GPSC2 (associated with PCV13 serotype 1) was most common before PCV was introduced, whereas GPSC3 (associated with non-PCV13 serotype 8) predominated after PCV13 implementation. The emergence among non-PCV13 serotypes, of lineages (such as GPSC5/CC172 and GPSC9/63) that are usually associated with PCV13 serotypes, warrants continued genomic surveillance in South Africa, more so as PCV10 (Pneumosil), which excludes serotypes 3, 4 and 18C, is currently being rolled out in South Africa to replace PCV13.

## Supplementary material

10.1099/mgen.0.001559 Uncited Supplementary Material 1.

10.1099/mgen.0.001559 Uncited Supplementary Material 2.

## Abbreviations

CC: clonal complex
CI: confidence interval
DMP: diagnostic media products
GPSC: global pneumococcal sequence cluster
IPD: invasive pneumococcal disease
IRR: incidence rate ratio
MDR: multidrug resistance
NVT: non-vaccine-type
PBP: penicillin-binding protein
PCV: pneumococcal conjugate vaccine
VT: vaccine-type
WGS: whole-genome sequencing
