# Supplementary material for: Population snapshot of Streptococcus pneumoniae causing invasive disease among adults aged ≥18 years in South Africa before and after implementation of pneumococcal conjugate vaccines in 2005–2020
Source: Microb Genom. 2025 Nov 19;11(11):001559. doi: 10.1099/mgen.0.001559 (PMC12668798; doi:10.1099/mgen.0.001559)
Supplement: Uncited Supplementary Material 1. [file mgen-11-01559-s001.pdf]

Supplementary Table 1. Changes in incidence rates of GPSC's in South Africa (pre-PCV (2005-2008) versus PCV7 (2009-2010) period)

GPSC	model	pre-PCV estimated cases	PCV7 estimated cases	pre-PCV average incidence/100 000 population	PCV7 average incidence/100 000 population	Average IRR_	95% CI lower limit	95% CI upper limit	Adjusted average p value
1	none	274	82	0.222	0.125	0.569	0.386	0.838	0.0069
2	negative bionomial	1747	997	1.412	1.523	1.075	0.945	1.222	0.2803
3	negative bionomial	731	305	0.592	0.466	0.784	0.632	0.972	0.0376
5	negative bionomial	477	86	0.385	0.131	0.341	0.241	0.483	<0.001
10	none	212	45	0.171	0.069	0.392	0.238	0.644	<0.001
13	negative bionomial	375	45	0.304	0.069	0.221	0.139	0.351	<0.001
14	none	83	300	0.067	0.458	6.738	4.268	10.638	<0.001
16	none	187	127	0.151	0.194	1.285	0.882	1.872	0.228
17	negative bionomial	673	436	0.549	0.667	1.224	1.001	1.497	0.065
18	none	141	173	0.114	0.264	2.318	1.565	3.434	<0.001
22	none	244	45	0.197	0.069	0.340	0.209	0.554	<0.001
26	none	83	173	0.067	0.264	3.863	2.398	6.225	<0.001
27	none	165	41	0.133	0.063	0.460	0.270	0.785	0.007
33	none	212	182	0.171	0.278	1.620	1.154	2.273	0.009
41	none	217	86	0.176	0.131	0.751	0.503	1.121	0.215
51	none	273	41	0.221	0.063	0.277	0.169	0.457	<0.001
54	none	242	45	0.196	0.069	0.346	0.212	0.564	<0.001
56	none	113	214	0.091	0.327	3.605	2.378	5.465	<0.001
70	none	218	259	0.176	0.395	2.271	1.654	3.119	<0.001
79	none	153	45	0.124	0.069	0.546	0.323	0.923	0.038

93	none	116	86	0.094	0.131	1.399	0.873	2.240	0.215

GPSC, Global Pneumococcal Sequence Cluster. Significant p-value (<0.05). Lineages that increased significantly are highlighted.

Supplementary Table 2. Changes in incidence rates of GPSC’s in South Africa (pre-PCV (2005-2008) versus early-PCV13 (2011-2014) period)

GPSC	model	pre-PCV estimated cases	early-PCV13 estimated cases	pre-PCV average incidence/100 000 population	early-PCV13 average incidence/100 000 population	Average IRR_	95% CI lower limit	95% CI upper limit	Adjusted average p value
1	none	274	110	0.222	0.079	0.366	0.236	0.568	<0.001
2	negative bionomial	1747	742	1.412	0.538	0.378	0.319	0.449	<0.001
3	negative bionomial	731	867	0.592	0.621	1.054	0.865	1.283	0.676
5	negative bionomial	477	240	0.385	0.174	0.448	0.328	0.611	<0.001
7	none	148	91	0.12	0.065	0.552	0.328	0.929	0.042
8	none	34	110	0.027	0.079	3.11	1.417	6.823	0.005
9	none	128	95	0.104	0.068	0.666	0.393	1.131	0.179
10	none	212	151	0.171	0.109	0.637	0.42	0.966	0.050
11	none	67	99	0.054	0.071	1.307	0.706	2.42	0.500
12	none	107	61	0.087	0.044	0.494	0.263	0.928	0.044
13	negative bionomial	375	278	0.304	0.203	0.662	0.486	0.902	0.018
14	none	83	469	0.067	0.337	4.95	3.111	7.877	<0.001
16	none	187	23	0.151	0.017	0.113	0.048	0.265	<0.001
17	negative bionomial	673	576	0.549	0.416	0.762	0.61	0.951	0.028
18	none	141	40	0.114	0.029	0.254	0.126	0.513	<0.001
22	none	244	76	0.197	0.055	0.277	0.165	0.463	<0.001
26	none	83	525	0.067	0.377	5.543	3.497	8.786	<0.001

27	none	165	40	0.133	0.029	0.217	0.109	0.433	<0.001
32	none	37	214	0.03	0.154	5.331	2.632	10.797	<0.001
33	negative bionomial	212	186	0.172	0.133	0.771	0.52	1.145	0.266
37	none	156	87	0.126	0.063	0.501	0.297	0.845	0.018
41	none	217	23	0.176	0.017	0.099	0.042	0.229	<0.001
46	none	67	115	0.054	0.083	1.516	0.833	2.758	0.226
48	none	71	87	0.057	0.063	1.086	0.583	2.025	0.910
51	none	273	221	0.221	0.159	0.719	0.504	1.025	0.097
52	none	120	132	0.097	0.095	0.977	0.596	1.603	1
54	negative bionomial	242	0	0.198	0.003	0.015	0.002	0.105	<0.001
56	none	113	329	0.091	0.236	2.602	1.694	3.996	<0.001
61	none	144	61	0.116	0.044	0.37	0.203	0.676	0.002
68	none	153	112	0.124	0.08	0.655	0.402	1.067	0.142
70	none	218	424	0.176	0.305	1.744	1.257	2.421	0.002
77	none	83	101	0.067	0.073	1.058	0.592	1.89	0.910
79	none	153	63	0.124	0.045	0.374	0.209	0.671	0.001
93	none	116	59	0.094	0.042	0.46	0.246	0.857	0.025

GPSC, Global Pneumococcal Sequence Cluster. Significant p-value (<0.05). Lineages that increased significantly are highlighted.

Supplementary Table 3. Changes in incidence rates of GPSC's in South Africa (pre-PCV (2005-2008) versus late-PCV13 (2015-2020) period)

GPSC	model	pre-PCV estimated cases	Late-PCV13 estimated cases	pre-PCV average incidence/100 000 population	Late-PCV13 average incidence/100 000 population	Average IRR_	95% CI lower limit	95% CI upper limit	Adjusted average p value
1	none	274	90	0.222	0.039	0.179	0.102	0.313	<0.001
2	negative bionomial	1747	68	1.412	0.031	0.020	0.011	0.037	<0.001
3	negative bionomial	731	1548	0.592	0.680	1.145	0.947	1.384	0.231

4	none	77	38	0.062	0.017	0.256	0.102	0.642	0.004
5	negative bionomial	477	255	0.385	0.113	0.287	0.202	0.407	<0.001
6	none	86	55	0.070	0.024	0.332	0.153	0.722	0.007
7	none	148	146	0.120	0.064	0.527	0.315	0.881	0.022
9	none	128	214	0.104	0.094	0.914	0.568	1.471	0.763
10	none	212	355	0.171	0.155	0.904	0.624	1.310	0.744
11	none	67	58	0.054	0.025	0.478	0.219	1.043	0.115
12	none	107	80	0.087	0.035	0.391	0.202	0.758	0.010
13	negative bionomial	375	98	0.304	0.043	0.138	0.081	0.235	<0.001
14	none	83	125	0.067	0.055	0.812	0.444	1.487	0.675
16	none	187	389	0.151	0.170	1.123	0.772	1.635	0.698
17	negative bionomial	673	885	0.549	0.390	0.715	0.574	0.892	0.006
18	none	141	34	0.114	0.015	0.139	0.059	0.331	<0.001
21	none	79	67	0.064	0.029	0.447	0.214	0.932	0.045
22	none	244	107	0.197	0.047	0.240	0.142	0.405	<0.001
26	none	83	570	0.067	0.250	3.674	2.290	5.894	<0.001
27	none	165	17	0.133	0.007	0.059	0.018	0.192	<0.001
30	negative bionomial	34	179	0.027	0.078	3.046	1.396	6.643	0.006
32	negative bionomial	37	240	0.030	0.106	3.610	1.752	7.439	<0.001
33	negative bionomial	212	197	0.172	0.086	0.506	0.327	0.781	0.005
34	none	49	111	0.040	0.049	1.218	0.587	2.529	0.763
37	none	156	25	0.126	0.011	0.083	0.030	0.233	<0.001
38	none	114	90	0.092	0.039	0.435	0.232	0.815	0.014
41	none	217	0	0.179	0.003	0.015	0.002	0.107	<0.001
46	none	67	136	0.054	0.060	1.099	0.587	2.057	0.911
48	none	71	92	0.057	0.040	0.677	0.341	1.343	0.391
49	none	49	111	0.040	0.049	1.218	0.587	2.529	0.763
51	negative bionomial	273	405	0.222	0.178	0.812	0.580	1.137	0.311
52	none	120	213	0.097	0.093	0.975	0.600	1.582	1
54	none	242	55	0.196	0.024	0.122	0.060	0.245	<0.001

56	negative bionomial	113	454	0.093	0.201	2.204	1.429	3.400	0.001
57	negative bionomial	40	89	0.033	0.039	1.218	0.547	2.712	0.763
61	none	144	132	0.116	0.058	0.496	0.292	0.844	0.018
68	none	153	32	0.124	0.014	0.107	0.042	0.271	<0.001
70	negative bionomial	218	359	0.178	0.159	0.902	0.625	1.303	0.744
79	none	153	13	0.124	0.006	0.043	0.010	0.177	<0.001
92	negative bionomial	0	99	0.003	0.046	13.807	1.837	103.746	0.001
93	none	116	65	0.094	0.028	0.308	0.154	0.617	0.002
125	negative bionomial	0	91	0.003	0.043	12.995	1.723	97.985	0.002
137	negative bionomial	49	240	0.039	0.104	2.707	1.420	5.160	0.004
142	none	77	42	0.062	0.018	0.299	0.126	0.712	0.009
155	none	37	70	0.030	0.031	1.083	0.456	2.570	1
162	none	79	28	0.064	0.012	0.203	0.076	0.541	0.001
168	none	40	43	0.032	0.019	0.569	0.216	1.494	0.425
178	none	0	167	0.003	0.076	23.553	3.208	172.899	<0.001
Unknown	none	0	91	0.003	0.042	12.995	1.723	97.985	0.002

GPSC, Global Pneumococcal Sequence Cluster. Significant p-value (<0.05). Lineages that increased significantly are highlighted

Supplementary Table 4. GPSC's expressing 2 or more serotypes, South Africa, pre-PCV (2005-2008), PCV7 (2009-2010), early-PCV13 (2011-2014) and late-PCV13 (2015-2020) period

GPSC (N)	Serotype* (n)	2005-2008 No. of samples	2009-2010 No. of samples	2011-2014 No. of samples	2015-2020 No. of samples
3 (194)	**3 (1)**	0	0	0	1
	8 (154)	15	4	23	112
	31 (2)	0	0	1	1
	11A (1)	0	0	0	1
	15A (3)	0	0	0	3
	22F (24)	2	2	2	18
	15B (1)	0	0	0	1
	33F (8)	1	1	2	4
5 (45)	**19A (7)**	4	1	2	0
	**19F (2)**	0	0	0	2
	23A (1)	0	0	0	1
	23B (3)	0	0	0	3
	**23F (8)**	7	0	1	0
	35B (15)	0	0	3	12
	**6A (5)**	1	0	3	1
	7C (3)	0	1	0	2
	9N (1)	0	0	0	1
9 (24)	**14 (8)**	3	0	3	2
	15A (16)	0	0	1	15
10 (41)	**3 (18)**	0	0	2	16
	**14 (13)**	5	1	2	5
	10A (6)	0	0	0	6
	**19A (2)**	0	0	0	2
	**19F (1)**	0	0	0	1
	serogroup 24 (1)	0	0	0	1
13 (28)	**6A (27)**	9	8	1	9
	**6B 1)**	0	0	0	1
14 (33)	**19A (1)**	0	0	1	0
	**23F (31)**	2	7	14	8
	**6B (1)**	0	0	1	0
16 (43)	13 (7)	1	0	0	6
	**23F (1)**	0	0	0	1
	**6A (2)**	0	0	1	1
	9N (27)	4	3		20
	serogroup 24 (6)	0	0	0	6
17 (122)	**19A (120)**	16	10	19	75
	**19F (2)**	0	0	0	2
30 (23)	10A (7)	0	0	0	7
	33D (5)	1	1	1	2
	35B (1)	0	0	0	1
	**6A (1)**	0	1	0	0
	**6C (9)**	0	0	0	9

32 (31)	12F (4)	1	0	0	3
	**7F (27)**	0	2	8	17
33 (31)	16F (30)	6	3	6	15
	**19A (1)**	0	1	0	0
56 (56)	12F (55)	3	5	12	35
	**7F (1)**	0	0	1	0
61 (20)	**3 (1)**	0	0	0	1
	17F (16)	3		2	11
	18A (2)	1	0	0	1
	22F (1)	0	0	0	1

*PCV13 serotypes are bolded

Supplementary Table 5. Antimicrobial susceptibly profiles (percentages) of common lineages

GPSC (n)	Penicillin % (n)	Chloramphenicol % (n)	Erythroycin (ermB) % (n)	Erythtromycin (mefA) % (n)	Tetracyclin % (n)	Co-trimoxazole % (n)	MDR % (n)
**1 (20)**	100 (20)	0	95 (19)	95 (19)	95 (19)	100 (20)	95.0 (19)
2 (97)	0	8.3 (8)	0	0	8.3 (8)	13.4 (13)	0
3 (194)	0	0.5 (1)	0	0	0	3.6 (7)	0
**5 (45)**	91.1 (41)	0	4.5 (2)	11.4 (5)	4.5 (2)	91.1 (41)	15.9 (2)
7 (21)	0	0	0	0	0	42.9 (9)	0
**9 (26)**	100 (26)	7.7 (2)	69.2 (18)	0	100 (26)	46.2 (12)	72.0
**10 (42)**	100 (42)	0	34.1 (14)	0	100 (42)	100 (42)	34.2
13 (28)	25.9 (7)	0	0	0	0	67.9 (19)	0
**14 (34)**	85.3 (29)	11.8 (4)	29.4 (10)	70.6 (24)	100 (34)	100 (34)	84.9
16 (43)	7.0 (3)	4.7 (2)	2.3 (1)	4.7 (2)	7 (3)	14 (6)	7.0
**17 (124)**	97.6 (121)	0	0	0	0.8 (1)	98.4 (122)	1.3
26 (76)	1.3 (1)	96.0 (73)	0	0	100 (76)	100 (76)	4.4
30 (23)	0	0	0	0	0	91.3 (21)	0
32 (31)	0	0	0	0	0	0	0
33 (31)	22.6 (7)	0	0	0	0	48.4 (15)	0
46 (19)	0	0	0	0	0	52.6 (10)	0
51 (52)	0	0	0	0	0	0	0
52 (27)	0	0	0	0	0	0	0
56 (54)	0	0	0	0	0	14.8 (8)	0
61 (20)	5 (1)	0	0	0	0	10 (2)	0
70 (58)	0	0	0	0	0	0	0
137 (27)	0	0	0	0	0	15.4 (4)	0
178 (21)	0	0	0	0	0	76.2 (16)	0
